# Preparation and Thermal Performance Study of a Novel Organic–Inorganic Eutectic Phase Change Material Based on Sodium Acetate Trihydrate and Polyethylene Glycol for Heat Recovery

**DOI:** 10.3390/ma18010164

**Published:** 2025-01-03

**Authors:** Wanchun Sun, Xuyan Xu, Tao Zhang, Zhijiang Wu, Yansheng Xu

**Affiliations:** 1 School of Energy and Automotive Engineering, Shunde Polytechnic, Foshan 528300, China; 23088@sdpt.edu.cn (T.Z.); 10669@sdpt.edu.cn (Z.W.); 10222@sdpt.edu.cn (Y.X.); 2 Department of Industrial Engineering, Tsinghua University, Beijing 100084, China; xuxy19@mails.tsinghua.edu.cn

**Keywords:** phase change material, sodium acetate trihydrate, polyethylene glycol, organic–inorganic eutectic PCM, expanded graphite

## Abstract

A novel organic–inorganic eutectic phase change material (PCM) based on sodium acetate trihydrate (SAT) and polyethylene glycol (PEG) was developed to meet the needs of heat recovery and building heating. Three kinds of PEG with different molecular weights were selected to form organic–inorganic eutectic PCM with SAT. The thermal properties of three series of SAT-PEG eutectic PCM were compared based on DSC results, focusing on the impact of PEG addition on the phase change temperature and enthalpy of SAT, as well as the melting uniformity. The inhibitory effects of two nucleating agents on the supercooling of SAT-PEG eutectic PCM were systematically investigated. The effect of PEG on the crystallization behavior of SAT was studied using a metallographic microscope. To evaluate the thermal reliability of the SAT-PEG eutectic PCM, 600 cycles of melting–solidification experiments were conducted. Experimental results show that SAT can form eutectic PCMs with PEG200, PEG600, and PEG6000, respectively, with high enthalpy and excellent melting uniformity. The phase change temperature ranged from 55 °C to 60 °C and the enthalpy was as high as 250–280 kJ/kg. The results of the cooling curves show that 10 wt% tetrasodium pyrophosphate decahydrate (TPD) can reduce the supercooling degree to less than 1 °C. Significantly, all three series of SAT-PEG eutectic PCMs exhibit exceptional thermal reliability after 600 cycles of melting–solidification, with shifts in the phase change temperatures and enthalpies of less than 4%. XRD diffraction patterns showed that SAT, PEG, and TPD were physically mixed without a chemical reaction to form new substances. Microscopic images reveal that the addition of PEG preserves the original needle-shaped crystal morphology of SAT while reducing its crystal size. The rapid formation of small crystals can provide more nucleation points and expedite crystallization, thereby enhancing the heat release capabilities of the PCM.

## 1. Introduction

According to comprehensive statistics, global energy consumption is showing a sustained upward trend [1]. However, the reliance on fossil fuels is no longer sufficient to meet the continuously increasing energy demand. It is widely recognized that the reserves of fossil fuels are depleting rapidly, and their combustion process significantly pollutes the environment [2]. Consequently, the utilization of renewable energy has received great attention from the scientific community and the industrial sector due to its environmentally friendly and pollution-free characteristics compared to fossil fuels [3]. Currently, the utilization of renewable energy faces challenges such as high initial costs and low efficiency, making it imperative to focus on improving utilization rates and reducing energy waste. In both developed and developing countries, the operations of buildings account for over 40% of global energy consumption and contribute to one-third of global greenhouse gas emissions [4]. Most of this energy is used for HVAC systems to provide comfortable indoor thermal environments, stable hot water supply, and hygienic living conditions for occupants [5]. In order to get on track with the Net Zero Emissions of the building sector, it is imperative to enhance the efficiency of renewable energy sources such as solar energy, biomass, and geothermal energy or utilize medium-temperature waste heat from the industrial sector for building heating. These strategies will effectively reduce reliance on fossil fuels, promote energy conservation in the building sector, and significantly mitigate carbon emissions.

Thermal energy storage (TES) technology based on phase change materials (PCMs) offers an efficient solution to the spatial and temporal disparities in heat distribution [6]. PCMs possess the capacity to absorb and release heat via the process of melting and solidification within a specific temperature range during phase transition, allowing for the effective storage and transportation of thermal energy. Nowadays, PCMs are widely used in indoor heating and cooling [7], refrigerated transport and distribution applications [8], transportation [9], the thermal management of electronics [10], photovoltaic/thermal (PV/T) systems [11], heat pump systems [12,13], and so on. Common PCMs can be classified into two major categories: organic and inorganic. In the low-temperature application range (0–80 °C), organic PCMs represented by paraffin exhibit advantages such as suitable phase change temperature, high phase change enthalpy, and exceptional thermal reliability [14]. However, careful consideration must be given to safety concerns and economic costs when applying PCMs to buildings. Despite the aforementioned advantages of paraffin, its flammability and high cost constrain its widespread utilization [15]. On the other hand, inorganic PCMs represented by inorganic hydrated salts have the advantages of low cost, non-flammability, and high enthalpy [16]. For space heating and hot water supply in buildings, PCMs with a phase change temperature within the range of 50–60 °C are required. Among the inorganic hydrated salts meeting the aforementioned phase change temperature requirements, sodium acetate trihydrate (SAT) shows good development prospects due to its appropriate phase change temperature, high phase change enthalpy (271.09 kJ/kg), cost-effectiveness, and non-flammability [17]. However, the published literature indicates that SAT has a high supercooling degree of up to 75 °C and severe phase separation, leading to a considerable reduction in its service life [18,19]. Adding nucleating agents and thickeners is a common approach to solving the above problems [20]. Li et al. [21] prepared a composite PCM based on SAT with carboxymethylcellulose (CMC) and disodium hydrogen phosphate dodecahydrate (DHPD) as thickeners and nucleating agents to solve the phase separation and supercooling problems. The experimental results showed that when the contents of CMC and DSP were both 4 wt%, the supercooling degree of the composite PCM was only 0.96 °C. Hua et al. [22] compared the supercooling suppression effects of six nucleating agents on SAT, including disodium hydrogen phosphate (DSP), tetrasodium pyrophosphate decahydrate (TPD), anhydrous sodium acetate (SAA), sodium tetraborate decahydrate (STD), and sodium metasilicate nonahydrate (SMN). It was found that DSP could be an effective nucleating agent for SAT. When the addition of DSP was 2 wt%, the supercooling degree of SAT was reduced to about 2 °C. And 1–1.5 wt% of xanthan gum (XG) could effectively suppress the phase separation of SAT. Furthermore, Wang et al. [23] demonstrated experimentally that TPD could effectively mitigate the supercooling phenomenon of SAT. On the other hand, the incorporation of nanoparticles, such as carbon nanotubes [24], nano copper [25], AlN nanoparticles [26], Ti_2_O_3_ [27], and α-Al_2_O_3_, has also demonstrated efficacy in mitigating the supercooling of SAT, with the minimum value reaching 0.4 °C [28].

In addition, taking SAT as the main phase change material and then introducing a small amount of organic matter to construct an organic–inorganic eutectic phase change system is also a method to optimize the thermal properties of SAT. Wu et al. [29] developed a novel composite PCM composed of SAT, acetamide, and micron/nano-aluminum nitride. They found that acetamide could co-melt with SAT and reduce the melting point of SAT to meet the need of heat storage in solar heat pump systems. The above composite PCM exhibited a high latent heat of 222.6 kJ/kg. Fu et al. [30] investigated the thermal properties of an organic–inorganic eutectic mixture composed of SAT and urea. They found that the addition of 8% urea could make the SAT-urea mixture have a suitable phase change temperature (47.84 °C) and maintain high latent heat (223.1 kJ/kg), while reducing the supercooling temperature to 1.54 °C. In our previous research work [31], an SAT-urea-DHPD eutectic phase change system was constructed and its thermophysical properties were studied, including the effects of urea on the melting consistency, phase change temperature, and enthalpy of SAT. It was found that the addition of urea could effectively improve the melting uniformity of SAT and help to improve the phase separation phenomenon. In other words, appropriate organic PCMs can fully leverage their benefits of consistent melting behavior and exceptional thermal reliability to collaboratively establish a co-melting phase change system with inorganic hydrated salt SAT, thereby overcoming the inherent drawbacks of SAT such as significant supercooling and serious phase separation.

In the current work, a novel organic–inorganic hybrid PCM system composed of SAT and polyethylene glycol (PEG) was developed. PEG is an oligomer (molecular weight below 20,000) with a broad range of phase change temperatures (−55 °C to 100 °C), dependent on its molecular weight. It offers the advantages of high phase change enthalpy, excellent thermal stability, absence of phase separation, and non-toxicity [32,33]. Significantly, its high water solubility contributes to optimizing the co-melting effect of organic and inorganic phases. PEG200, PEG600, and PEG6000 were selected to construct organic–inorganic eutectic PCMs with inorganic hydrated salt SAT. The effect of PEG addition on the phase change thermal properties of SAT was systematically investigated, including phase change temperature, enthalpy, and melting uniformity. The influence of PEG with different molecular weights on the thermal properties of SAT was compared. Next, based on the measurement results of the cooling curves, the nucleating agents for the novel SAT-PEG eutectic PCM were screened and their addition amounts were optimized. The investigated nucleating agents included DHPD and TPD. In order to study the crystallization behavior of SAT-PEG eutectic PCM more clearly, its crystal morphology and size were characterized using a metallographic microscope. In addition, expanded graphite was introduced as a porous carrier to adsorb the SAT-PEG eutectic PCM to prepare the composite PCM. The microstructures of expanded graphite and composite materials were characterized via scanning electron microscopy. Finally, 600 melting solidification experiments were conducted on the SAT-PEG eutectic PCM to further investigate the improvement effect of organic phase PEG on the thermal reliability of SAT.

## 2. Materials and Methods

### 2.1. Materials and Reagents

Sodium acetate trihydrate (SAT, CH_3_COONa·3H_2_O, AR), Polyethylene glycol 6000 (PEG6000, AR), disodium hydrogen phosphate dodecahydrate (DHPD, Na_2_HPO_4_·12H_2_O, AR), and tetrasodium pyrophosphate decahydrate (TPD, Na_4_P_2_O_7_·10H_2_O, AR) were purchased from Tianjin Kemiou Chemical Reagent Co., Ltd. (Tianjin, China). Polyethylene glycol 200 (PEG200, AR) and Polyethylene glycol 600 (PEG600, AR) were purchased from China National Pharmaceutical Group Chemical Reagent Co., Ltd. (Shanghai, China). Expanded graphite (EG, 100 meshes) was purchased from Qingdao Furuite graphite Co., Ltd. and Dao Technologies Ltd. (Qingdao, China).

### 2.2. Preparation

#### 2.2.1. SAT-Polyethylene Glycol (PEG) Eutectic PCM

The SAT-PEG eutectic PCM was prepared using the mixed heating method. The specific steps were as follows: Firstly, SAT and PEG were weighed accurately according to the preset proportion and placed in a reagent bottle. Then, the temperature of the blast drying oven was set at 70 °C, and the reagent bottle was put in after the cap was tightened for heating. When 80% of the samples in the reagent bottle melted into a liquid state, the reagent bottle was taken out and placed in a water bath at 70 °C for stirring. The melting of the samples was accelerated by means of water bath heating and stirring. Finally, when no solid particles were observable in the reagent bottle, the nucleating agent was added. Under continuous stirring and heating, the PCM sample achieved a uniformly molten state, and the SAT-PEG eutectic PCM was obtained.

#### 2.2.2. SAT-Polyethylene Glycol (PEG)/Expanded Graphite Composite PCM

The preparation steps of the composite PCM consisting of the SAT-PEG eutectic PCM and expanded graphite were as follows: Firstly, the eutectic PCM was placed in a blast drying oven at 70 °C for heating, while the weighed expanded graphite was placed into a high-temperature-resistant PE bag. Then, the molten eutectic PCM was poured into the PE bag, and a glass rod was employed to uniformly blend the PCM and the expanded graphite. During the mixing process, if the eutectic PCM solidified, the PE bag was sealed properly and placed in the blast drying oven for reheating and then mixing was resumed. The above steps were repeated until no eutectic PCM was observable on the wall of the PE bag, and the SAT-PEG/expanded graphite composite PCM was obtained.

### 2.3. Characterization

The phase change thermophysical properties of the samples were characterized using a differential scanning calorimeter (DSC). The specific steps are as follows: firstly, 8–12 mg of the sample was weighed using an analytical balance. The container for loading the sample was an aluminum crucible matching the measurement equipment (NETZSCH, DSC214, Shanghai, China). For liquid samples, a disposable dropper was used for aspiration, and for solid samples, a stainless steel spoon was used for scooping. Next, the crucible with the sample was sealed and placed at a specific position in the measurement chamber. Then, the thermophysical properties of the sample were measured under a nitrogen atmosphere. The temperature measurement range was 0–90 °C, and the heating rate was 5 °C/min [34,35,36]. The flow rates of nitrogen purge gas and guard gas were 40 mL/min and 60 mL/min, respectively. Finally, once the measurement was completed, the data file was loaded into DSC 214 Proteus 1.0 software for analysis to read the thermophysical property data such as the phase change temperature and enthalpy value.

The structure of the sample was characterized using X-ray diffraction patterns (XRD, Rigaku Smart Lab SE, Tokyo, Japan). The crystals structure of the SAT-PEG sample was characterized using a metallographic microscope (DM2700M, Leica, Wetzlar, German). The microstructures of expanded graphite and composite PCMs were characterized using a scanning electron microscope (TESCAN MIRA LMS, Teskin (China) Co., Ltd., Shanghai, China).

### 2.4. Evaluation of Supercooling Degree and Thermal Reliability

**Figure 1:**
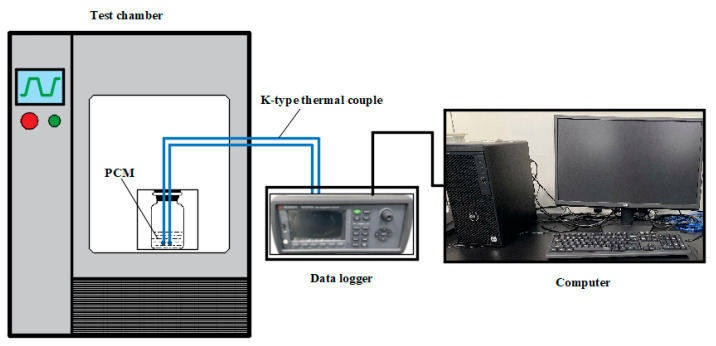
Schematic diagram of the experimental apparatus for measuring the cooling curve.

The experimental setup for measuring the cooling curve of the sample is depicted in Figure 1. The measurement equipment includes a high-low temperature alternating damp heat test chamber (HT-SC-80B, Huitai Machine CO., Ltd., Dongguan, China), a data acquisition instrument (Keysight DAQ970A, Santa Rosa, CA, USA), and a computer. Among them, the test chamber is used to control the ambient temperature of the sample. The data acquisition instrument measures and collects the temperature data of the sample through K-type thermocouples and uploads the temperature data to the computer. The measurement steps are as follows: firstly, 50 g of the sample was weighed and placed in a reagent bottle; two K-type thermocouples were installed prior to tightening the bottle cap. Then, the sample was placed in the test chamber, and the chamber temperature changed in accordance with the set procedure. During the experiment, the chamber temperature was first heated from room temperature to 80 °C, maintained at this temperature for 1 h to ensure the complete melting of the sample, and then cooled from 80 °C to 0 °C, maintained at this temperature for 1 h to ensure the complete solidification of the sample. Both the heating and cooling rates were 1 °C/min. Finally, the temperature data were exported from the analysis software on the computer and organized to obtain the cooling curve diagram of the sample.

The melting–solidification cycling test was also performed by controlling the chamber temperature to realize the repeated melting and solidification of the sample. In the cycling experiment, the weight of each sample was controlled within 10–15 g to achieve rapid melting and solidification. The temperature control program of the test chamber was set as follows: the chamber temperature was heated from room temperature to 80 °C at a rate of 1.5 °C/min, maintained at 80 °C for 20 min, and then cooled from 80 °C to 0 °C at a rate of 1.5 °C/min and maintained at 0 °C for 20 min.

The precision of the instruments and the uncertainties of the measured parameters during characterization are listed in Table 1, which were analyzed in our previous work [31]. The uncertainties of the measured parameters were analyzed based on the Evaluation and Expression of Measurement Uncertainty (JJF1059-2012) [37,38]. For the parameters directly measured by the operator (the geometry dimension of the test room and the supercooling degree of the samples), the uncertainties were calculated using the Type A evaluation method. The calculation formula is $u_{A}(\bar{x})=\sqrt{\frac{1}{N(N-1)}\sum_{i=1}^{N}{\left(x_{i}-\bar{x}\right)}^{2}}$ , where *x_i_* and $\bar{x}$ are the measured and average values of the parameters, respectively, and *N* is the number of measurements. For the parameters measured by the instrument, the uncertainties are calculated using the Type B evaluation method (uB=ak, where *a* is the instrument precision; *k* is the coverage factor and its value is $\sqrt{3}$).

## 3. Results and Discussion

### 3.1. Phase Change Properties of SAT-Polyethylene Glycol Eutectic PCMs

**Figure 2:**
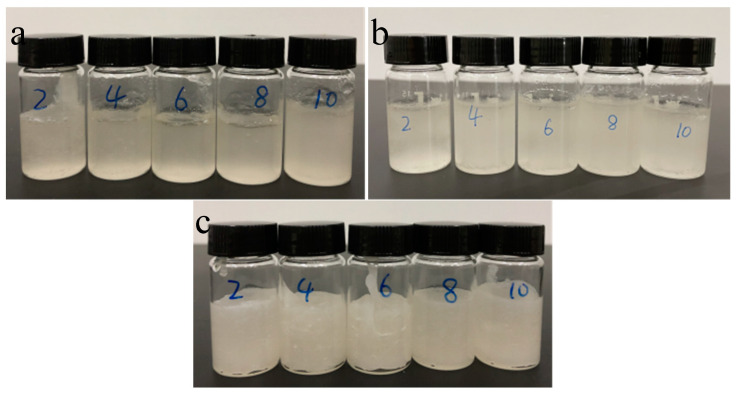
Images of the organic–inorganic SAT-PEG eutectic PCM in the molten state (at 70 °C): (**a**) SAT-PEG200; (**b**) SAT-PEG600; (**c**) SAT-PEG6000.

**Figure 3:**
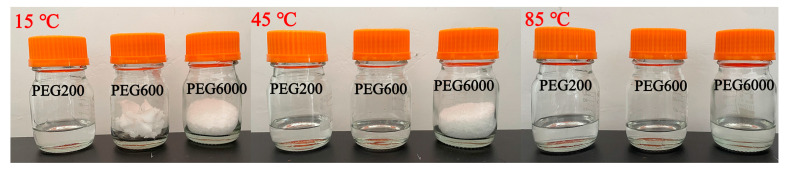
Photographs of PEG200, PEG600, and PEG6000 samples at different ambient temperatures (15 °C, 45 °C, and 85 °C).

**Figure 4:**
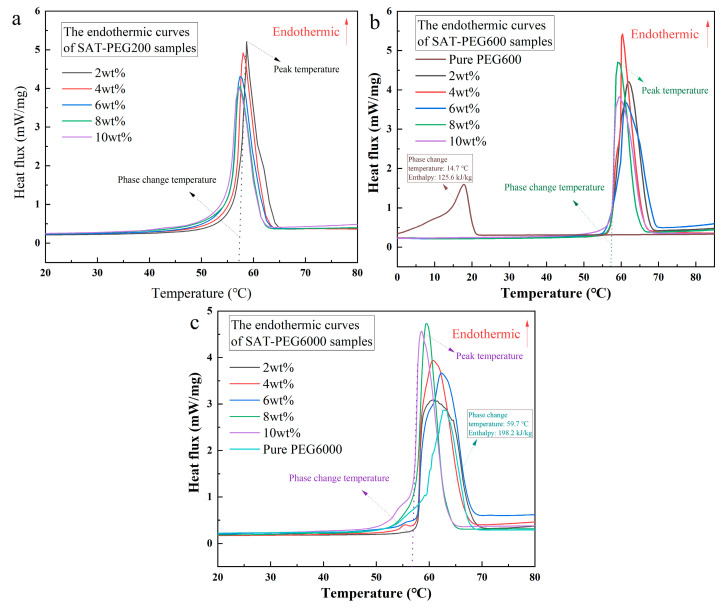
DSC curves of the organic–inorganic eutectic PCMs of (**a**) SAT-PEG200, (**b**) SAT-PEG600, and (**c**) SAT-PEG6000.

In order to improve the melting homogeneity of SAT, PEG200, PEG600, and PEG6000 were adopted as organic phases to construct organic–inorganic eutectic PCMs with inorganic phase SAT, respectively. The organic–inorganic eutectic PCMs were prepared by heating and mixing organic and inorganic components. The eutectic PCMs are referred to as SAT-PEG200, SAT-PEG600, and SAT-PEG6000, respectively. The sample images of the SAT-PEG eutectic PCMs are shown in Figure 2. The numbers written on the reagent bottles in the picture indicate the mass fraction of PEG. Due to the addition of PEG200, PEG600, and PEG6000, inorganic hydrated salt SAT shows improved homogeneity in the molten state, and its transparency decreases with the increase in the molecular weight of PEG. This phenomenon is related to the phase change temperature of PEG: with the increase in molecular weight, the phase change temperature of PEG gradually increases. Photographs of the PEG200, PEG600, and PEG6000 samples at different ambient temperatures (15 °C, 45 °C, and 85 °C) are shown in Figure 3. Owing to the fact that the phase change temperature of PEG200 is about −65 °C, the DSC instrument used in this study cannot satisfy the measurement conditions. The DSC curves of PEG600 and PEG6000 used in the experiments are presented in Figure 4. At a room temperature of 15 °C, PEG200 and PEG600 are in a liquid state while PEG6000 is a waxy solid. As the molecular weight increases, the viscosity of PEG also increases. Consequently, in the SAT-PEG eutectic phase-change system, employing a higher molecular weight PEG will progressively augment the enhancement effect on the uniformity of the sodium acetate system, but the fluidity will gradually diminish.

The DSC test results of the SAT-PEG eutectic PCM samples in three series after the first melting–solidification cycle are presented in Figure 4, with detailed data listed in Table 2. As depicted in Figure 4, all three molecular weight PEG200, PEG600, and PEG6000 can form a eutectic with SAT and maintain the phase change temperature within the range of 50–60 °C. In Figure 4a, it is evident that an increase in the amount of added PEG200 leads to a gradual leftward shift of the phase change peak for the SAT-PEG200 eutectic PCM. This trend is consistent with the thermal property data provided in Table 2, where both the phase change temperature and enthalpy of SAT-PEG200 eutectic PCM exhibit a decreasing trend. Similar observations hold true for SAT-PEG600 and SAT-PEG6000 eutectic PCM. Based on the data analysis presented in Table 2, it is evident that the addition of PEG200, PEG600, and PEG6000 has a negligible effect on reducing the phase change enthalpy of SAT. Even at a high concentration of 6 wt% for PEG6000, the resulting SAT-PEG6000 eutectic PCM still exhibits a substantial enthalpy value of 253.7 kJ/kg. It can be concluded that the incorporation of organic phase PEG can effectively mitigate the inherent limitations of SAT phase separation and enhance its practical utility. In order to further optimize the thermal performance of the SAT-PEG eutectic PCM, a selection of nucleating agents and determination of their addition amount are carried out based on the SAT-PEG200 (6 wt%) eutectic PCM in the next section.

### 3.2. Selection of Nucleating Agents for the SAT-PEG Eutectic PCMs

**Figure 5:**
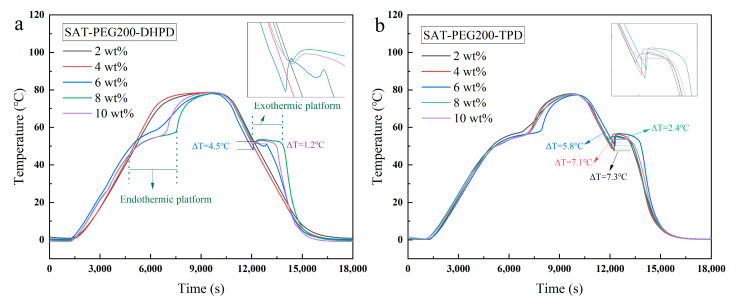
Cooling curves of the SAT-PEG 200 eutectic PCM containing the nucleating agent of (**a**) DHPD and (**b**) TPD.

Taking SAT-PEG200 (6 wt%) as the base eutectic PCM, the influences of two nucleating agents, including disodium hydrogen phosphate dodecahydrate (DHPD) and tetrasodium pyrophosphate decahydrate (TPD), on the supercooling degree of the eutectic PCM were investigated. The cooling curves are shown in Figure 5 and the specific data are listed in Table 3.

**Figure 6:**
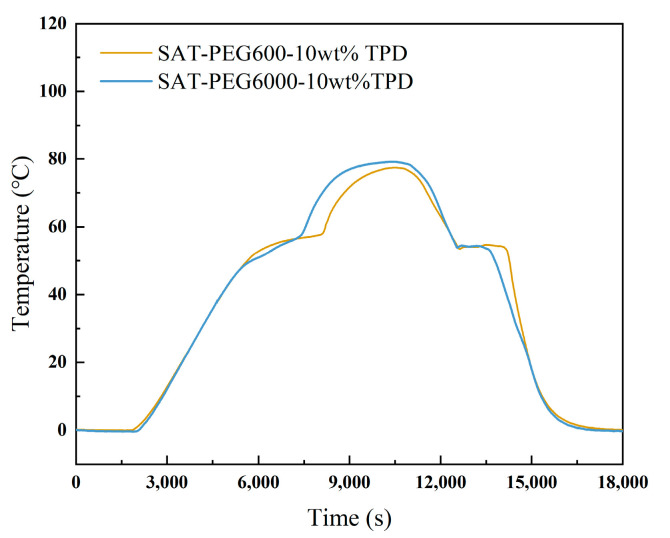
Cooling curves of the SAT-PEG600 and SAT-PEG6000 eutectic PCMs containing nucleating agent of 10 wt% TPD.

Based on the experimental results presented in Figure 5 and Table 3, it can be seen that TPD shows a better supercooling degree inhibition effect. When the mass fraction of the nucleating agent attains 10 wt%, the supercooling degree of SAT-PEG200 is as low as 0.1 °C. To verify the inhibitory effect of TPD on the other two eutectic PCMs, namely SAT-PEG600 and SAT-PEG6000, cooling curve tests were conducted on the samples of the above two eutectic PCMs with 10 wt% TPD added. The addition amount of the organic phase remains at 6 wt%, and the results are depicted in Figure 6. As can be observed from Figure 6, TPD is capable of eliminating the supercooling degrees of both SAT-PEG600 and SAT-PEG6000 eutectic PCMs. Consequently, tetrasodium pyrophosphate decahydrate (TPD) was determined as the nucleating agent for the SAT-PEG series eutectic PCMs.

### 3.3. Microstructure of SAT-PEG Eutectic PCM

**Figure 7:**
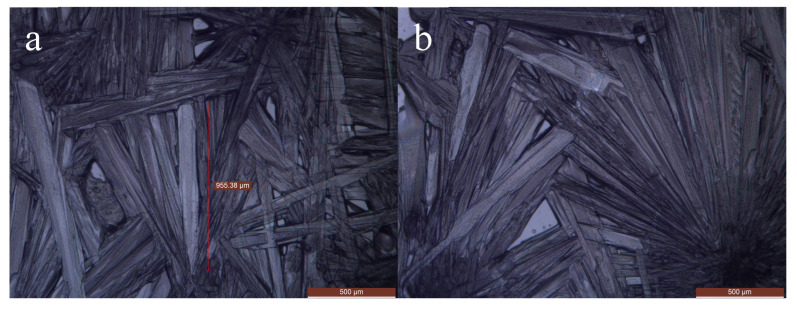
Microscopic images of SAT crystals With a scale of 500 μm (**a**,**b** represent sampled images from different locations).

**Figure 8:**
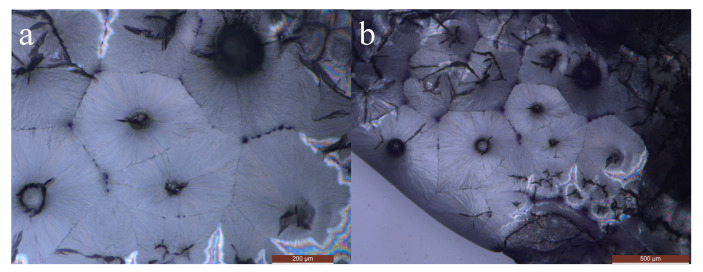
Microscopic images of PEG6000 crystals with a scale of (**a**) 200 μm; (**b**) 500 μm.

**Figure 9:**
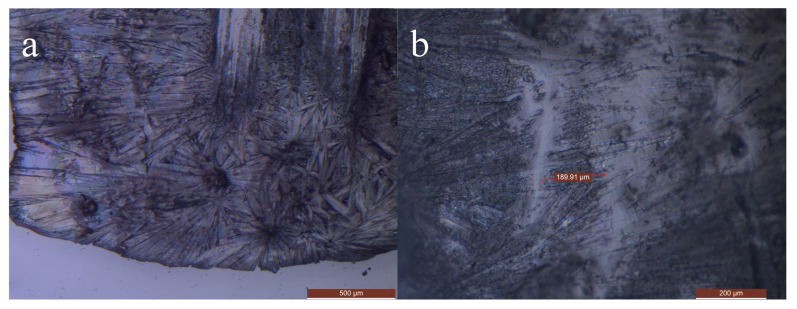
Microscopic images of SAT-PEG6000 eutectic PCM crystals with a scale of (**a**) 500 μm; (**b**) 200 μm.

In order to observe the crystal morphology of the SAT-PEG eutectic PCM, a metallographic microscope (DM2700M, Leica, Germany) was used to observe the crystals of SAT, PEG, and the organic–inorganic eutectic PCM. The SAT-PEG6000 eutectic PCM was selected as the characterization object. Figure 7 shows the crystals of sodium acetate trihydrate, which are needle shaped. During the crystallization process, the crystals interlace and overlap with each other, growing radially from the nucleation point. The crystal size is between 500 and 1000 μm. Figure 8 shows the crystals of PEG6000, whose crystal morphology is polygonal and clustered together under the microscope. Figure 9 shows the crystals of SAT-PEG6000 eutectic PCM. Since the addition amount of PEG6000 is only 6 wt%, the crystals of PEG6000 are uniformly dispersed among the crystals of SAT, as shown in Figure 8a,b. As shown in Figure 8b, a few polygonal crystals can be observed among a pile of needle-shaped crystals. The addition of PEG6000 does not change the original needle-shaped morphology of SAT. As can be seen from Figure 8a, the crystals of the SAT-PEG6000 eutectic PCM still exhibit a long needle-shaped form, but their size has changed, with the crystal size ranging from 200 to 500 μm. This indicates that the organic phase PEG6000 can reduce the crystal size of the inorganic hydrated salt SAT. The rapid formation of small crystals will provide more nucleation points for the crystallization of the hydrated salt SAT, thereby accelerating its crystallization rate. This is because the organic substance PEG with a larger crystal size is capable of providing a framework for the inorganic hydrated salt SAT and utilizing its relatively high electronegativity to pair with Na+ of the inorganic hydrated salt SAT, offering nucleation sites and reducing the interfacial energy, thereby facilitating the rapid generation of SAT crystals.

**Figure 10:**
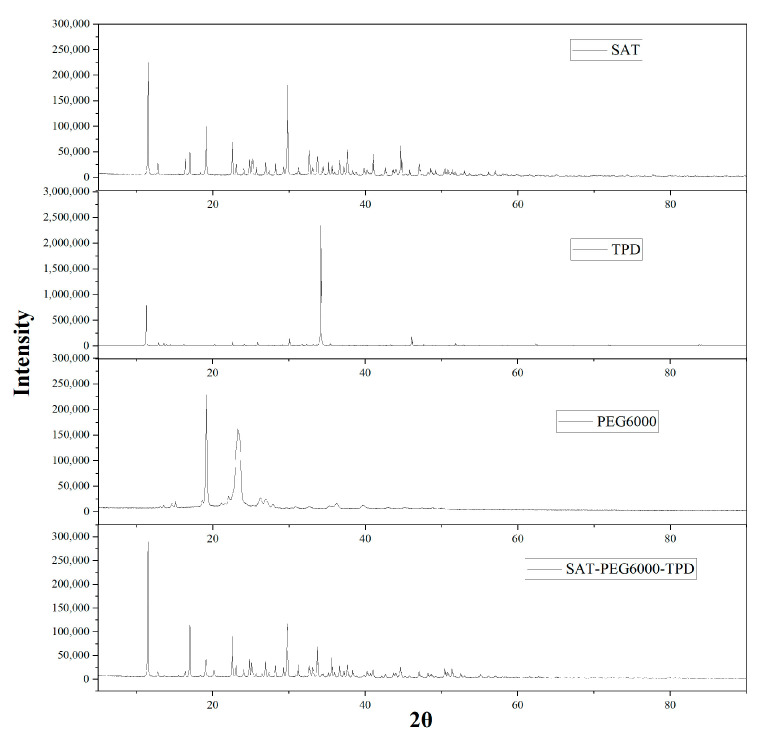
XRD diffraction patterns of SAT, PEG6000, TPD, and SAT-PEG6000-TPD eutectic PCM.

To further verify whether a chemical reaction occurred during the preparation of the SAT-PEG6000 eutectic PCM, X-ray diffraction characterization was carried out on the inorganic hydrated salt SAT, the nucleating agent TPD, the organic phase PEG6000, and the SAT-PEG6000 eutectic PCM. As shown in Figure 10, no new diffraction peaks were generated in the XRD pattern of the SAT-PEG6000 eutectic PCM. This implies that among SAT, TPD, and PEG6000, it is merely a physical blend and no chemical reaction occurs. This is in mutual correspondence with the phenomenon that the crystal form of the eutectic PCM observed under the metallographic microscope does not change.

### 3.4. Microstructure and Thermal Reliability of the Composite PCM

**Figure 11:**
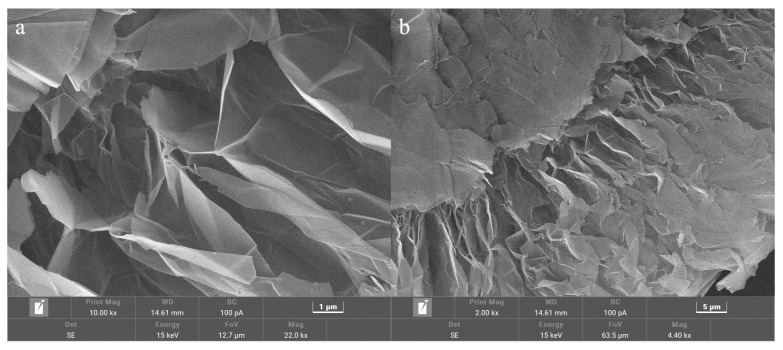
SEM images of 100-mesh expanded graphite with different magnification factors: (**a**) 10,000 times and (**b**) 2000 times.

**Figure 12:**
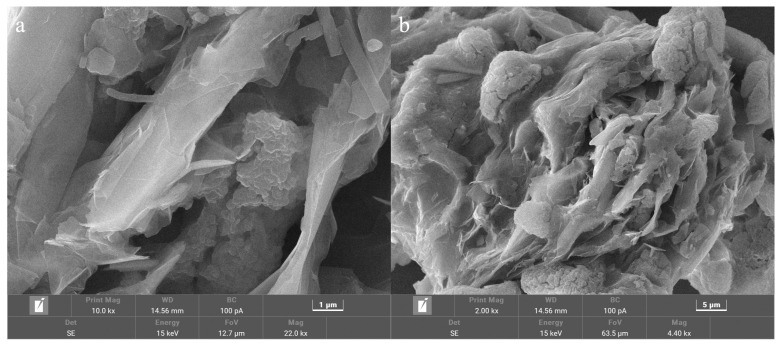
SEM images of SAT-PEG6000/expanded graphite composite PCM with different magnification factors: (**a**) 10,000 times and (**b**) 2000 times.

To further enhance the thermal conductivity of the SAT-PEG6000 eutectic PCM and meet the requirements for rapid heat storage and release applications in waste heat recovery systems, 100-mesh expanded graphite was used as a thermal conductive carrier and compounded with the SAT-PEG6000 eutectic PCM to prepare a carbon-based composite phase change material (PCM). As shown in Figure 11, a layered porous microstructure of expanded graphite can be observed under the scanning electron microscope. This provides a carbon-based carrier for the SAT-PEG6000 eutectic PCM and inhibits the fluidity of the material in the molten state, thereby expanding the application scope of the composite PCM. Figure 12 shows the SEM images of the SAT-PEG6000/expanded graphite composite PCM. It can be observed that the SAT-PEG6000 eutectic PCM nucleates and crystallizes within the pores of expanded graphite and ultimately fills the porous architecture of the expanded graphite. Owing to the constraint imposed by the pore structure of expanded graphite, the growth space for the phase change material crystals is restricted. Consequently, the crystals of the SAT-PEG6000 eutectic PCM exhibit the phenomenon of small crystals coalescing into blocks under the scanning electron microscope.

**Figure 13:**
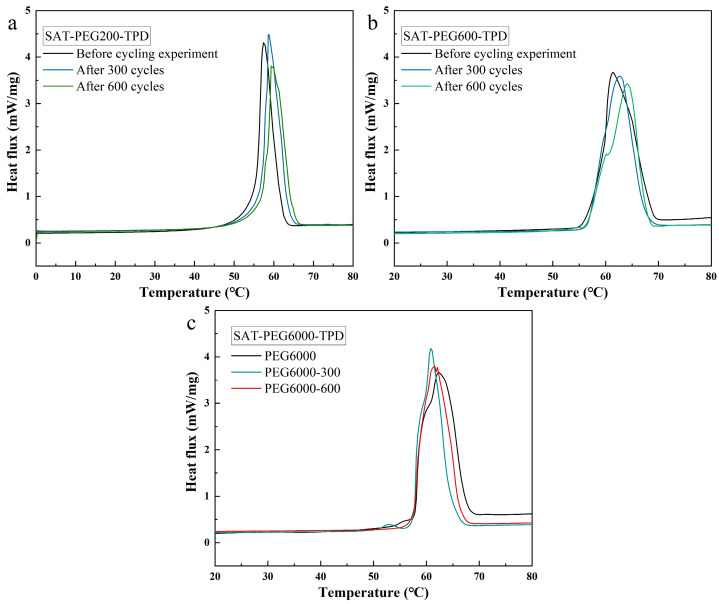
DSC results of three kinds of SAT-PEG eutectic PCMs before and after melting–solidification cycling experiments: (**a**) SAT-PEG200 eutectic PCM; (**b**) SAT-PEG600 eutectic PCM; (**c**) SAT-PEG6000 eutectic PCM.

In practical applications, the recycling performance of phase change materials is of great significance, as it determines the service life and application effect of the phase change heat storage module. Therefore, in this work, a high- and low-temperature humid heat cycling test chamber was employed to conduct 600 melting–solidification cycling experiments on the thermal reliability performance of the SAT-PEG eutectic PCMs. Moreover, the phase change temperature and enthalpy value of the eutectic PCMs after the cycling tests were tested using DSC. The DSC curves are shown in Figure 13, and the data are listed in Table 4. After conducting 600 melting–solidification experiments, all three types of SAT-PEG eutectic PCMs demonstrated remarkable cyclic thermal reliability: the phase change temperature of the SAT-PEG200 eutectic PCM shifted by 4.89%, and the enthalpy value decreased by 1.47%; the phase change temperature of the SAT-PEG600 eutectic PCM shifted by 0.69%, and the enthalpy value decreased by 9.60%; the phase change temperature of the SAT-PEG6000 eutectic PCM shifted by 0.52%, and the enthalpy value decreased by 5.35%.

Table 5 compares the phase change performance of different PCMs based on SAT, and the data are collected from reported references and this study. It can be seen that as the eutectic component of SAT, PEG can well preserve the initial phase change temperature and enthalpy of pure SAT. From an economic point of view, the use of expanded graphite, nano-copper, and nano-AlN in composite PCMs will significantly increase the material costs of PCM products. If nanoparticles are added to solve the supercooling phenomenon of SAT, they can be replaced with low-cost DHPD or TPD. However, the addition of carbon materials or metal nanoparticles can significantly improve the thermal conductivity of PCM composites. Therefore, it is reasonable to select and optimize the content of the eutectic composition of PCM according to the requirement of phase change temperature, enthalpy, thermal conductivity and the limitation of cost budget.

## 4. Conclusions

In this work, a novel eutectic PCM based on SAT and PEG with excellent phase change performance was developed for heat recovery and building heat supply. The effects of organic PEG on the phase change thermal properties, melting uniformity and crystal morphology of inorganic hydrated salt SAT were investigated using DSC, microscopic images, and XRD diffraction patterns. The nucleating agents for the novel eutectic PCM were selected and the optimal addition was determined using cooling curves. According to the experimental results and the above discussion, the following conclusions can be drawn.

1. Organic compound PEG can form a eutectic with inorganic hydrated salt SAT to construct an organic–inorganic eutectic PCM system with good melting homogeneity. This significantly improves the phase separation of SAT. Meanwhile, the phase change temperature of 55–60 °C and high enthalpy value of 250–280 kJ/kg of SAT are effectively retained.
2. For the novel SAT-PEG eutectic PCM, TPD demonstrates superior supercooling suppression compared to DHPD. The addition of 10 wt% TPD can effectively reduce the supercooling degree of the three SAT-PEG eutectic PCMs (PEG200, PEG600, and PEG6000) to within 1 °C.
3. Microscopic images show that the SAT-PEG eutectic PCM still maintains the needle-shaped crystal morphology of SAT, and the introduction of PEG can reduce the crystal size of SAT. The rapid formation of small crystals will provide more nucleation points for crystallization, accelerate the crystallization rate, and reduce crystallization resistance, which will contribute to the rapid heat release of PCM.
4. After 600 melting–solidification experiments, the SAT-PEG200, SAT-PEG600, and SAT-PEG6000 eutectic PCMs all showed excellent thermal reliability, and the shifts in phase change temperature and enthalpy were within 4%.

## Figures and Tables

**Table 1:** Instrument precision and uncertainties of measured parameters.

Instrument	Precision	Measured Parameter	Uncertainty
Differential scanning calorimeter (NETZSCH, DSC214, Shanghai, China)	±1%	Melting points and latent heats of PCM samples	±0.58% (Type B)
High-low alternate temperature chamber (HT-SC-80B, Huitai Machine Co., Ltd., Dongguan, China)	±1.5 °C	Ambient temperature when measuring the cooling curves	±0.87 °C (Type B)
Data logger (Keysight DAQ970A, Santa Rosa, CA, USA)	±0.5 °C	Supercooling degree of PCM samples	±0.10 °C (Type A)
		Temperature curve of each test point	±0.29 °C (Type B)

**Table 2:** DSC test results of SAT-PEG eutectic PCMs.

Eutectic PCMs	Mass Fraction of PEG (wt%)	Phase Change Temperature (°C)	Enthalpy (kJ/kg)
SAT-PEG200	2	57.1	247.7
	4	56.6	239.2
	6	55.6	234.7
	8	55.8	211.4
	10	55.2	210.9
SAT-PEG600	2	57.3	291.9
	4	59.9	279.7
	6	59.3	271.1
	8	58.8	261.2
	10	57.5	255.3
SAT-PEG6000	2	58.0	269.4
	4	57.9	268.1
	6	57.9	253.7
	8	57.4	239.7
	10	57.3	237.5

**Table 3:** Specific data of cooling curves.

Nucleating Agent	Mass Fraction (wt%)	Supercooling Degree (°C)
DHPD	2	-
	4	-
	6	4.5
	8	1.4
	10	1.2
TPD	2	7.3
	4	7.1
	6	5.8
	8	2.4
	10	0.1

**Table 4:** DSC test results of SAT-PEG eutectic PCMs before and after melting–solidification cycling experiments.

Eutectic PCMs	Phase Change Temperature (°C)			Enthalpy (kJ/kg)		
	Before	After 300 Cycles	After 600 Cycles	Before	After 300 Cycles	After 600 Cycles
SAT-PEG200 −10 wt% TPD	55.2	57.1	57.9	210.9	218.5	207.8
SAT-PEG600 −10 wt% TPD	57.5	57.2	57.9	255.3	236.8	230.8
SAT-PEG6000 −10 wt% TPD	57.3	57.4	57.6	237.5	203.4	224.8

**Table 5:** Comparison of the phase change performance of PCMs based on SAT.

PCM Composition	Phase Change Temperature (°C)	Enthalpy (kJ/kg)	The Number of Cycles in the Melting Solidification Experiment
SAT/CMC/Nano-Cu/NaCl	55.88	231.2	20
SAT/SiC/EG [25]	58	240.6	200
SAT/Acetamide/micron/nano AlN [29]	47.3	222.6	100
SAT/Urea/DHPD/CMC/EG/Carbon nanotubes [31]	55.8	180.1	300
SAT/Acetamide/EG/DHPD/CMC [39]	51.6	235.5	200
SAT/Nano MgO/CMC [40]	58	240.8	-
SAT-PEG200-TPD-CMC	55.2	210.9	600
SAT-PEG600-TPD-CMC	57.5	255.3	600
SAT-PEG6000-TPD-CMC	57.3	237.5	600

## Data Availability

The original contributions presented in this study are included in the article. Further inquiries can be directed to the corresponding author.
